# The role of posterior pallial amygdala in mediating motor behaviors in pigeons

**DOI:** 10.1038/s41598-021-03876-7

**Published:** 2022-01-10

**Authors:** Xinmao Tian, Yuhua Shi, Yifeng Zhang, Yijie Wang, Mengke Li, Han Cheng, Zhenlong Wang

**Affiliations:** School of Life Sciences, Zhengzhou University, No. 100 Science Avenue, Zhengzhou, 450001 Henan Province People’s Republic of China

**Keywords:** Motor control, Animal behaviour

## Abstract

The posterior pallial amygdala (PoA) is located on the basolateral caudal telencephalon, including the basal division of PoA (PoAb) and the compact division of PoA (PoAc). PoA plays a vital role in emotion regulation and is considered a part of the amygdala in birds. However, the regulatory functions responsible for motor behaviors and emotions between PoAb and PoAc are poorly understood. Therefore, we studied the structure and function of PoA by tract-tracing methods, constant current electrical stimulation, and different dopamine receptor drug injections in pigeons (*Columba livia domestica*). PoAb connects reciprocally with two nuclear groups in the cerebrum: 1) a continuum comprising the temporo–parieto–occipitalis, corticoidea dorsolateralis, hippocampus, and parahippocampalis areas and 2) rostral areas of the hemisphere, including the nucleus septalis lateralis and nucleus taeniae amygdalae. Extratelencephalic projections of PoAb terminate in the lateral hypothalamic nucleus and are scattered in many limbic midbrain regions. PoAb and PoAc mainly mediated the turning movement. In the ‘open-field’ test, D1 agonist and D2 antagonist could significantly reduce the latency period for entering into the central area and increase the residence time in the central area, whereas D1 antagonist and D2 agonist had the opposite effect. PoAb and PoAc are important brain areas that mediate turning behavior.

## Introduction

The arcopallium/posterior pallial amygdala complex, previously known as the archistriatum (A), is present in the avian posterior ventral telencephalon^1^. After the Avian Brain Nomenclature Forum held at Duke University in July 2002, the Forum renamed most of the structures in the archistriatum that shared premotor characteristics to arcopallium, and for the remaining parts of the archistriatum that were assumed to constitute the amygdala, the Forum appended amygdala to their names to distinguish them from the arcopallial nuclei^1^. The acropallium/posterior pallial amygdala complex plays an essential role in controlling fear reactions in birds^2–6^. However, limited information is available about the contribution of its subdivisions.

As parts of the acropallium/posterior pallial amygdala complex, the posterior pallial amygdala (PoA) comprises the basal division of PoA (PoAb) and the compact division of PoA (PoAc). At present, PoAc shows some similarities in the receptor expression profile of the mammalian lateral regions of the amygdala (BLA)^7^. However, **there are few studies on PoAb**^7–9^. Electrical stimulation of the BLA induces active avoidance behavior due to fear in rats^10^, while the **roles** of PoAb and PoAc in mediating motor behaviors of birds remains unknown. Hence, these issues will be discussed in the present study.

As a common bird in daily lives, homing pigeons (*Columba livia domestica*) have many outstanding abilities, including homing and good orientation discrimination, making them an ideal model for studying neurological mechanisms. In this study, in terms of the fiber connections of PoA, we defined the afferent and efferent projections of PoAb in the pigeon using cholera toxin subunit B (CTB) and biotinylated dextran amine (BDA). Concerning the function of PoA, we first investigated the effects of PoA on the mediation of motor behavior in pigeons via electrical stimulation. And then, we further studied whether PoAc mediated motor behaviors through emotions by microinjection different dopamine D1 and D2 receptor agonists and antagonists.

## Results

### Fiber connections of PoAb

#### CTB retrograde labeling

**Figure 1:**
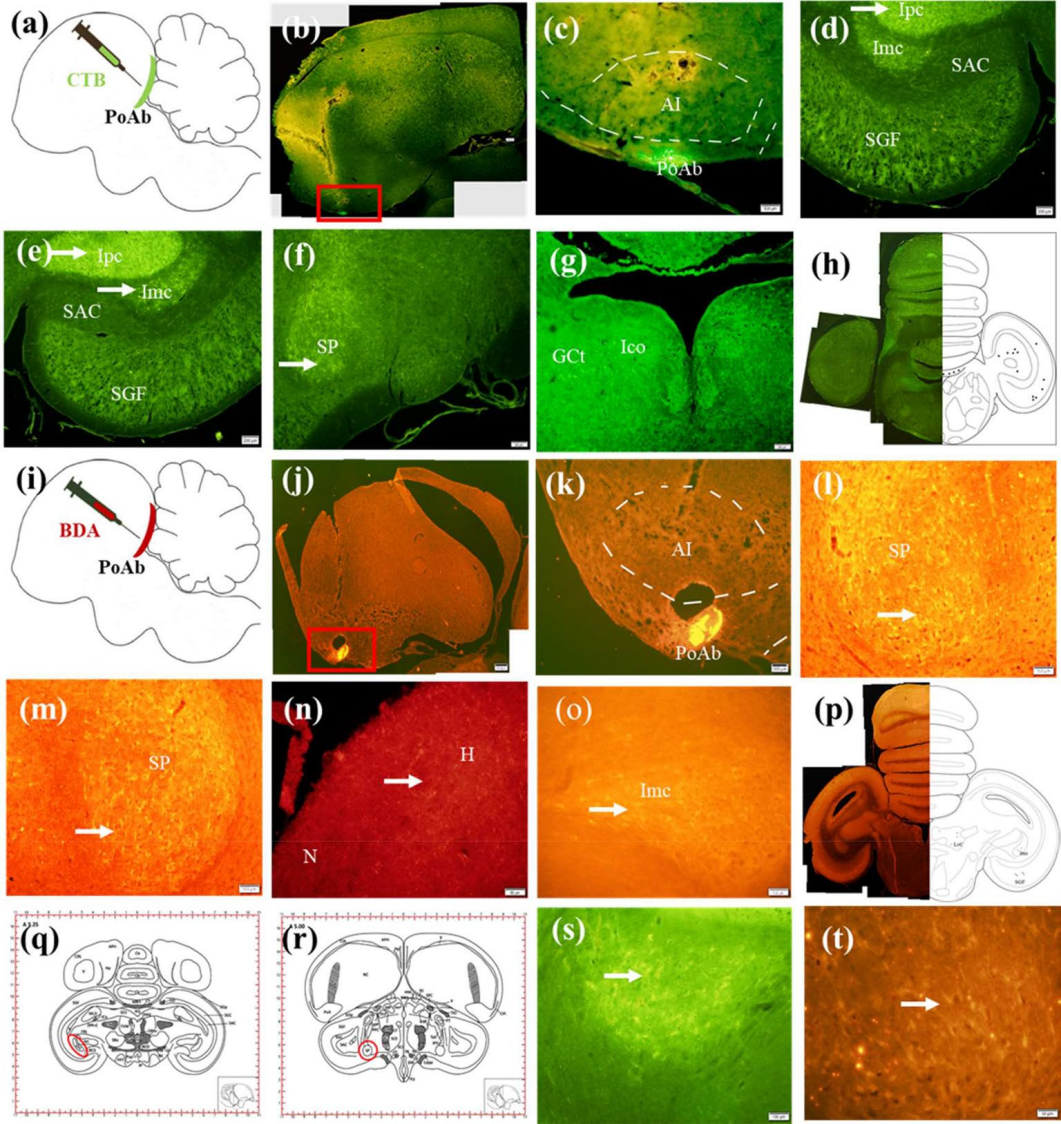
Fiber connections of PoAb. Schematic sagittal view of in vivo CTB injections (**a**) and BDA injections (**i**) into PoAb. (**b**) Injection site of CTB in the PoAb at A 6.25. (**c**) Enlargement of the box in (**b**). (**d**) Ipsilateral CTB-labeled neurons in Imc at A 3.5. (**e**) Contralateral CTB-labeled neurons in Imc at A 3.5. (**f**) Ipsilateral CTB-labeled neurons in SP at A 5.0. (**g**) Bilateral CTB-labeled neurons in GCt and Ico at A 3.25. (**h**) Schematic of CTB-labeled at A 1.25. (**j**) Injection site of BDA in the PoAb at A 5.75. (**k**) Enlargement of the box in (**j**). (**l**) Ipsilateral labeled fibers in SP at A 5.25. (**m**) Contralateral labeled fibers in SP at A 5.25. (**n**) Ipsilateral labeled fibers in N and H at A 13.0. (**o**) Contralateral labeled fibers in Imc at A 4.0. (**p**) Schematic of BDA-labeled at A 2.75. (**q**) Coronal whole brain map at A 3.25 based on Nissl staining results. (**r**) Coronal whole brain map at A 5.0 based on Nissl staining results. (**s**) Enlargement of the CTB-labeled neurons in Imc at A 3.25 in (**q**). (**t**) Enlargement of the BDA-labeled fibers in SP at A 5.25 in (**r**). (**b**–**h**) and (**j**–**t**) were the coronal slice of pigeon brain. Arrows indicate the labeled fibers and terminals, or the neurons. Scale bars = 200 µm in (**b**), (**d**), (**e**), (**f**), (**g**), (**h**), (**j**), (**k**), (**p**); 100 µm in (**c**), (**l**), (**m**), (**o**), (s**)**; 50 µm in (**n**), (**t**). *BaS* basorostral pallial nucleus; *BDA* biotinylated dextran amine; *CTB* cholera toxin subunit B; *GCt* substantia grisea centralis; *H* hyperpallium; *HA* hyperpallium apicale; *Ico* nucleus intercollicularis; *Imc* nucleus isthmi, pars magnocellularis; *Ipc* nucleus isthmi, pars parvocellularis; *N* nidopallium; *nIV* nucleus nervi trochlearis; *PoAb* basal division of posterior pallial amygdala; *SAC* stratum album central; *SGF* stratum griseum et fibrosum superficial; *SP* nucleus subpretectalis.

**Figure 2:**
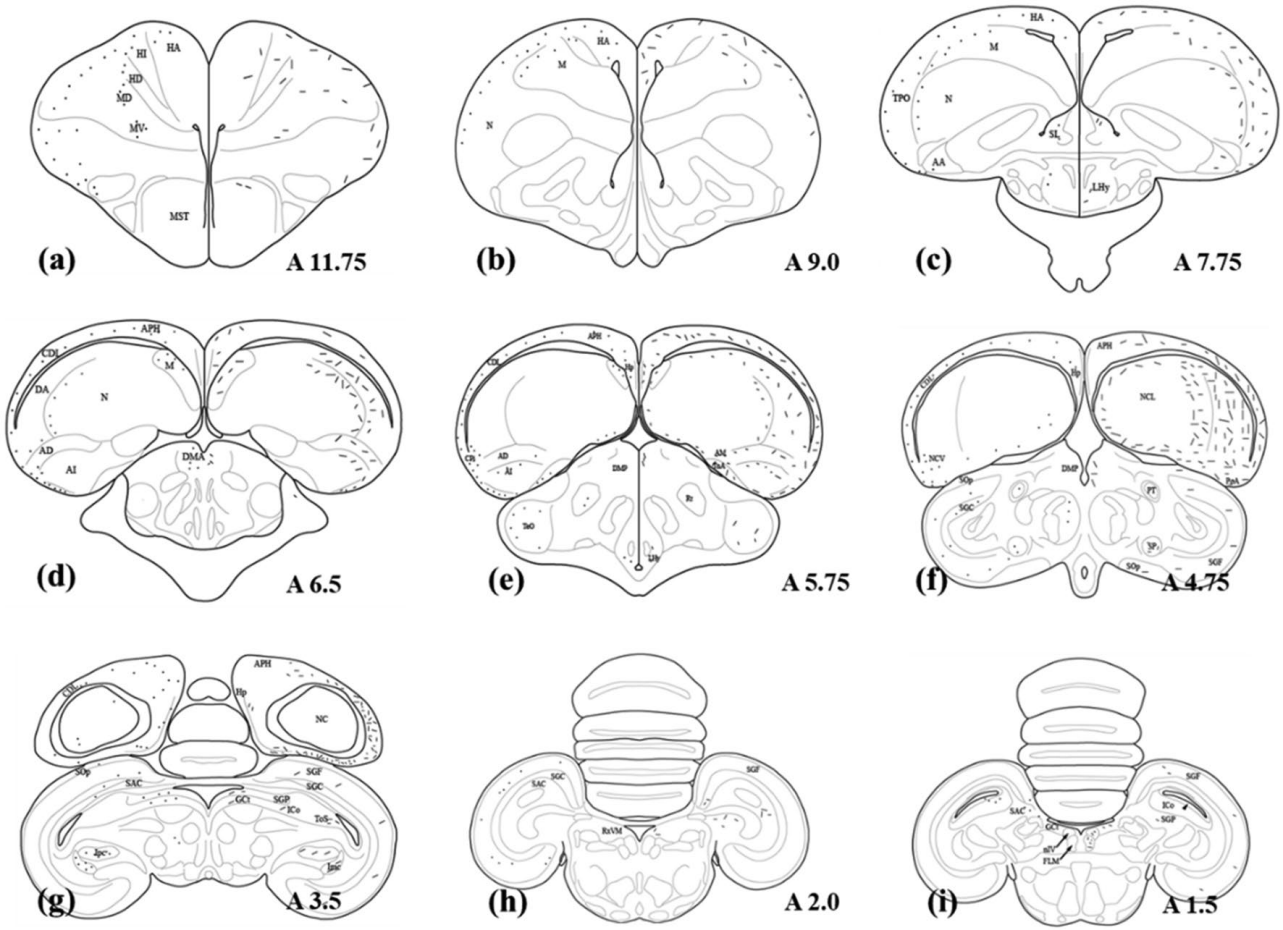
Schematic illustration of the rostrocaudal extent of labeling after BDA or CTB injections into the PoAb. Dots represent neurons retrogradely labeled with CTB on the left side, short lines represent anterogradely labeled terminals on the right side. Dots and lines indicate the relative differences in distribution but do not represent actual numbers. *BDA* biotinylated dextran amine; *CTB* cholera toxin subunit B; *PoAb* basal division of posterior pallial amygdala.

The center of the injection site of the right PoAb was placed at A 6.25, L 7.6, and D 6.8 according to the atlas of Pigeon^11^, and the diffusion of the tracer was mostly restricted within PoAb (Fig. 1b,c). Labeled areas outside PoAb included many regions of the forebrain. Around PoAb, numerous labelings were found in the cortex piriformis (CPI) from approximately A 5.00 to A 7.50, fewer labelings were found in the arcopallium dorsale (AD) and arcopallium intermedium (AI), and sparse labelings were found in the arcopallium anterius (AA) (Fig. 2c–f). Other labeled areas outside PoAb were divided into three streams in the telencephalon. Two of them were from the nidopallium caudoventrale, in which an extensively labeled area was found to be separated. One of them traveled dorsolaterally, along the way through the parahippocampalis (APH) and ventrolateral hippocampus (Hp) areas to the dorsolateral corticoid (CDL) and temporo–parieto–occipitalis (TPO) areas (Fig. 2c–g); in contrast, the other ran ventrally and turned rostrally, passing through the dorsolateral nidopallium caudolaterale (NCL), dorsal mesopallium (MD), and ventral mesopallium (MV). Labeled neurons proceeding rostrally extended to the hyperpallium apicale (HA), hyperpallium densocellulare, hyperpallium ventrale (HV), and hyperpallium intercalatum (HI) of the rostral telencephalon (Fig. 2a–c). They converged in the pallium at about A 7.0. The third course ran basal and turned rostrally. En route, it branched to nucleus taeniae amygdalae (TnA), nucleus septalis lateralis (SL) (Fig. 2c) and the anterior commissura (AC). Labeled neurons proceeding further rostrally extended to the basorostral pallial nucleus (BaS).

Numerous labeled neurons were observed in the nucleus dorsomedialis anterior thalami (DMA) (Fig. 2d–f) of the diencephalon. In contrast, fewer labeled neurons were observed in the radix mesencephalicus nervi trigemini (RxVM) (Fig. 2i), dorsolateral tectum opticum (TeO) (Fig. 2e) and nucleus preopticus anterior (POA). Sparsely distributed labeled neurons were observed in the lateral hypothalamic nucleus (LHy) (Fig. 2c) and nucleus rotundus.

In the midbrain, labeled neurons were widely scattered to a different site. CTB-labeled neurons were distributed in a continuous region stratum griseum central (SGC), stratum griseum et fibrosum superficiale (SGF) (Figs. 1d,e, 2g–i), nucleus isthmi, pars magnocellularis (Imc) (Fig. 1d,e,s), nucleus isthmi, pars parvocellularis (Ipc) (Fig. 1d,e), and formatio reticularis medialis mesencephali. Fewer labeled neurons were observed in the substantia grisea centralis (GCt) (Figs. 1g, 2g,h), nucleus intercollicularis (ICo) (Figs. 1g, 2g,h), substantia grisea et fibrosa periventricularis (SGP). Sparse labeling was observed in the locus ceruleus (LoC), nucleus pontis lateralis (PL), stratum album central (SAC) (Figs. 1d,e, 2h,i), nucleus subpretectalis (SP) (Figs. 1f, 2f), and ventral tegmental area (VTA).

#### BDA anterograde labeling

The injection of BDA to the right PoAb was located at A 5.75, L 7.60, D 6.10, and diffusion of the tracer was restricted within the nucleus (Fig. 1j,k). Similar to CTB-labeled neurons, around PoAb, many BDA-labeled fibers were found in CPI, AD, and AI. Moderate numbers of BDA-labeled fibers were observed in dorsolateral AA and arcopallium mediale (Fig. 2c–f). In contrast, PoAc contained few labeled fibers. Rostral to the injection, projections from PoAb were divided into four streams: one traveled dorsolaterally of the forebrain, along the way through APH and ventrolateral Hp to CDL and TPO, and among them, CDL extensively contained BDA-labeled fibers (Fig. 2c–g); in contrast, the other ran ventrally and turned rostrally, and the BDA-labeled fibers forming the continuum were found to exist extensively throughout NCL, MD, and MV. Labeled fibers extended to the HA, proceeding further rostrally. Less numerous labeled fibers existed in dorsolateral HV, and very sparsely labeled areas were observed in dorsolateral HI of the rostral telencephalon (Figs. 1m, 2a–c). The first two streams joined in the pallium, similar to the findings observed for CTB labeling. The third stream ascending fiber bundle extended rostrally toward the basal forebrain, and on this pathway, many fibers appeared in TnA (Fig. 2e). Moderate numbers of fibers appeared in SL and tuberculum olfactorium, and more rostrally, terminated in AC at approximately A 7.75. Using AC as a turning point, the contralateral bundle repeated its ipsilateral in a mirror or symmetric fashion. The fourth stream ran through the diencephalon and midbrain via a descending fiber bundle.

In the diencephalon, the main course of the descending pathway was primarily through the following areas: DMA and nucleus dorsomedialis posterior thalami (DMP) in the rostral diencephalon (Fig. 2e,f); then TeO and POM in the caudal diencephalon; and finally, a limited number of fibers in nIV, LHy, and RxVM.

In the midbrain, the BDA-labeled areas were much more than the CTB-labeled areas. The descending pathway labeled many areas along its course, and the labeled areas were divided into two streams. Ventrally, it projected massively to the limbic midbrain. There were several labeled varicose fibers or terminals in fasciculus longitudinalis medialis, SGC, SGF, and SP (Figs. 1l,m,t, 2g–i). In addition, BDA-labeled neurons were frequently observed in GCt, SAC, SGP, and Sop (Fig. 2g–i). The other labeled fibers were scattered in the midbrain, and these areas received a moderate number of labeled fibers from the descending pathway, including Imc (Fig. 1o,p), Ipc, OM and LoC (Fig. 1p). Few labeled fibers were distributed sparsely in torus semicircularis, VTA, and tractus vestibule–mesencephalicus (Supplmentary Fig S1 and Video S1).

**Figure 3:**
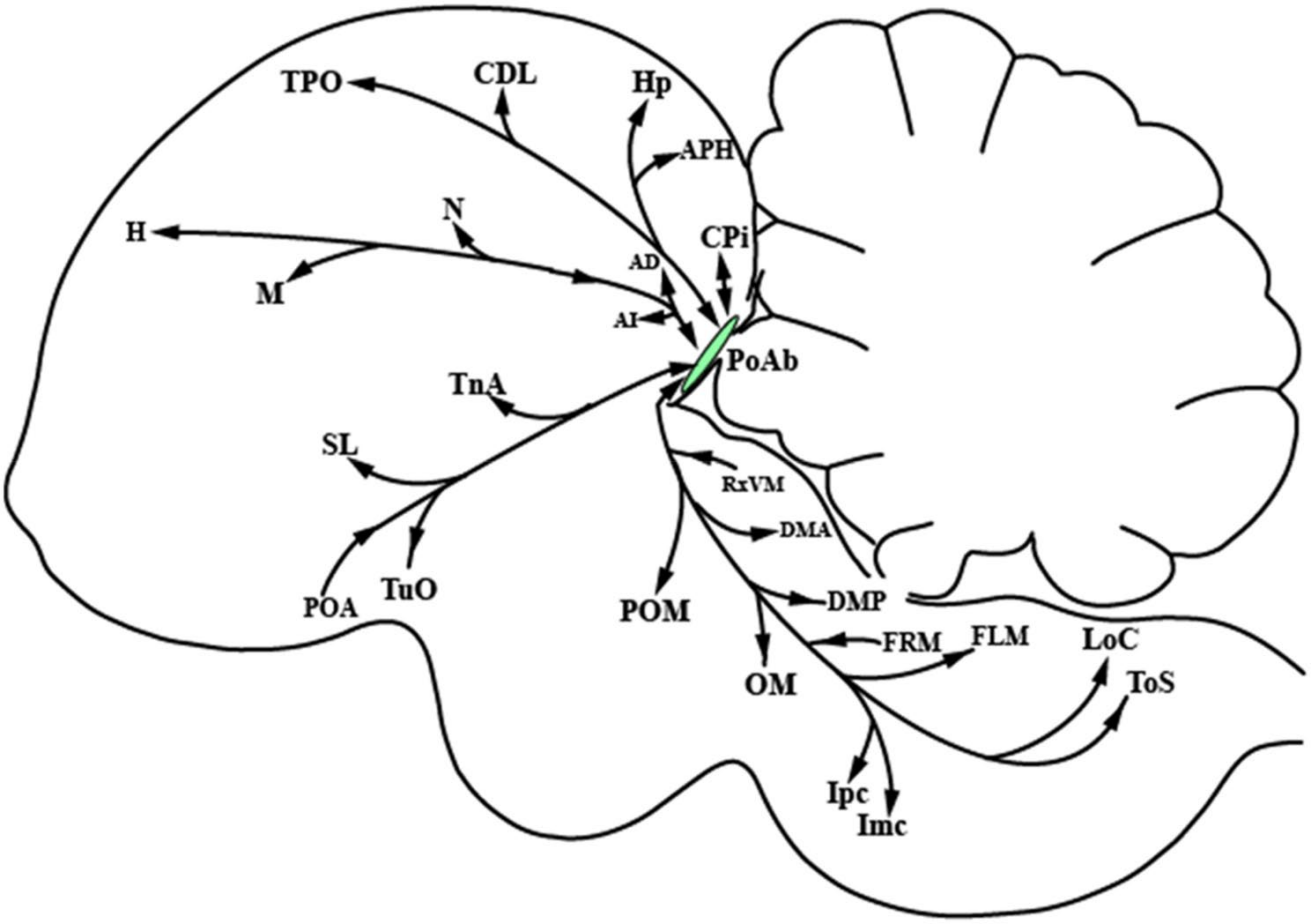
Models of main pathways of PoAb in the pigeon brain. Arrowheads at end of arrows show reciprocal connections, whereas those present midway indicate one-way projections. *AD* arcopallium dorsale; *AI* arcopallium intermedium; *APH* area parahippocampalis; *CDL* area corticoidea dorsolateralis; *CPi* cortex piriformis; *DMA* nucleus dorsomedialis anterior thalami; *DMP* nucleus dorsomedialis posterior thalami; *FLM* fasciculus longitudinalis medialis; *FRM* formatio reticularis medialis mesencephali; *H* hyperpallium; *Imc* nucleus isthmi, pars magnocellularis; *Ipc* nucleus isthmi, pars parvocellularis; *LoC* locus ceruleus; *M* mesopallium; *N* nidopallium; *OM* tractus occipito-mesencephalicus; *POA* nucleus preopticus anterior; *PoAb* basal division of posterior pallial amygdala; *POM* nucleus preopticus mediali; *RxVM* radix mesencephalicus nervi trigemini; *SL* nucleus septalis lateralis; *SP* nucleus subpretectalis; *TnA* nucleus taeniae amygdalae; *TPO* area temporo–parieto–occipitalis; *Tuo* tuberculum olfactorium.

In the CTB and BDA injections, three pigeons each were were targeted to PoAb. In each case, two were largely confined to the PoAb and with successful labeling, so they were selected to describe and depict the results. Figures 1–3 and Table 1 represent composites results. The general patterns of the afferent and efferent projections of PoAb are summarized in Fig. 3 and Table 1.

**Table 1:** Distribution of labeling of BDA and CTB injection in PoAb.

Sites	Number of CTB—labeled neurons		Density of BDA—labeled fibers and terminals	
	Ipsilateral	Contralateral	Ipsilateral	Contralateral
**Telencephalon**				
AA (arcopallium anterius)	+	−	+ +	−
AC (anterior commissura)	+ + +	+ + +	+ + +	+ + +
AD (arcopallium dorsale)	+ +	+ +	+ + +	+
AI (arcopallum intermedium)	+ +	+ +	+ + +	+
AM (arcopallium mediale)	−	−	+ +	−
APH (area parahippocampalis)	+ + +	+ + +	+ + +	+ + +
BaS (basorostral pallial nucleus)	+	+	−	−
CDL (area corticoidea dorsolateralis)	+ + +	+ + +	+ + +	+ + +
CPi (cortex piriformis)	+ + +	+ + +	+ + +	+ + +
HA (hyperpallium apicale)	+ +	+	+ + +	+ + +
HD (hyperpallium densocellulare)	+ +	+	−	−
HI (hyperpallium intercalatum)	+	−	+ +	−
Hp (hippocampus)	+ +	+ +	+ + +	+
HV (hyperpallium ventrale)	−	−	+	+
MD (mesopallium dorsale)	+ + +	+ + +	+ + +	+ + +
MV (mesopallium ventrale)	+ + +	+ + +	+ + +	+ + +
NCL (nidopallium caudolaterale)	+ + +	+ + +	+ + +	+ + +
NCV (nidopallium caudoventrale)	+ + +	+ + +	+ +	+ +
SL (nucleus septalis lateralis)	+ +	+ +	+ +	+ +
TnA (nucleus taeniae amygdalae)	+	+	+ + +	−
TPO (area temporo–parieto–occipitalis)	+ + +	−	+ + +	−
Tuo (tuberculum olfactorium)	−	−	+ +	+ +
**Diencephalon**				
DMA (nucleus dorsomedialis anterior thalami)	+ +	+ + +	+ + +	+ + +
DMP (nucleus dorsomedialis posterior thalami)	−	−	+ +	+ +
nIV (nucleus nervi trochlearis)	−	−	+	+
LHy (lateral hypothalamic nucleus)	+	+	+	+
POA (nucleus preopticus anterior)	+ +	+ +	−	−
POM (nucleus preopticus mediali)	−	−	+ +	+ +
Rt (nucleus rotundus)	+	+	−	−
RxVM (radix mesencephalicus nervi trigemini)	+ +	+ +	+	+
TeO (tectum opticum)	+ +	+ +	+ +	+ +
**Midbrain**				
FLM (fasciculus longitudinalis medialis)	−	−	+ + +	+ + +
FRM (formatio reticularis medialis mesencephali)	+ + +	+ + +	−	−
GCt (substantia grisea centralis)	+ +	+ +	+ +	+ +
Ico (nucleus intercollicularis)	+ +	−	−	−
Imc (nucleus isthmi, pars magnocellularis)	+ + +	+ + +	+ +	+ +
Ipc (Nucleus isthmi, pars parvocellularis)	+ + +	+ + +	+ +	+ +
LoC (locus ceruleus)	+	+	+ +	+ +
OM (tractus occipito-mesencephalicus)	−	−	+ +	+ +
PL (nucleus pontis lateralis)	+	+	−	−
PT (nucleus pretectalis)	−	+	−	−
SAC (stratum album central)	+	+	+ +	+ +
SGC (stratum griseum central)	+ + +	+ + +	+ + +	+ + +
SGF (stratum griseum et fibrosum superficia)	+ + +	+ + +	+ + +	+ + +
SGP (substantia grisea et fibrosa periventricularis)	+ +	−	+ +	+ +
Sop (stratum opticum)	−	−	+ +	+ +
SP (nucleus subpretectalis)	+	+	+ + +	+ + +
SpL (nucleus spiriformis lateralis)	−	+	−	−
ToS (torus semicircularis)	−	−	+	+
TVM (tractus vestibule–mesencephalicus)	−	−	+	+
VTA (ventral tegmental area)	+	+	+	+

Number of labeled neurons and density of labeled fibers and terminals: +  +  + , numerous; +  + , moderate; + , few; − , absent.

*BDA* biotinylated dextran amine; *CTB* cholera toxin subunit B; *PoAb* basal division of posterior pallial amygdala.

### The effects of electrical stimulation on motor behaviors

**Figure 4:**
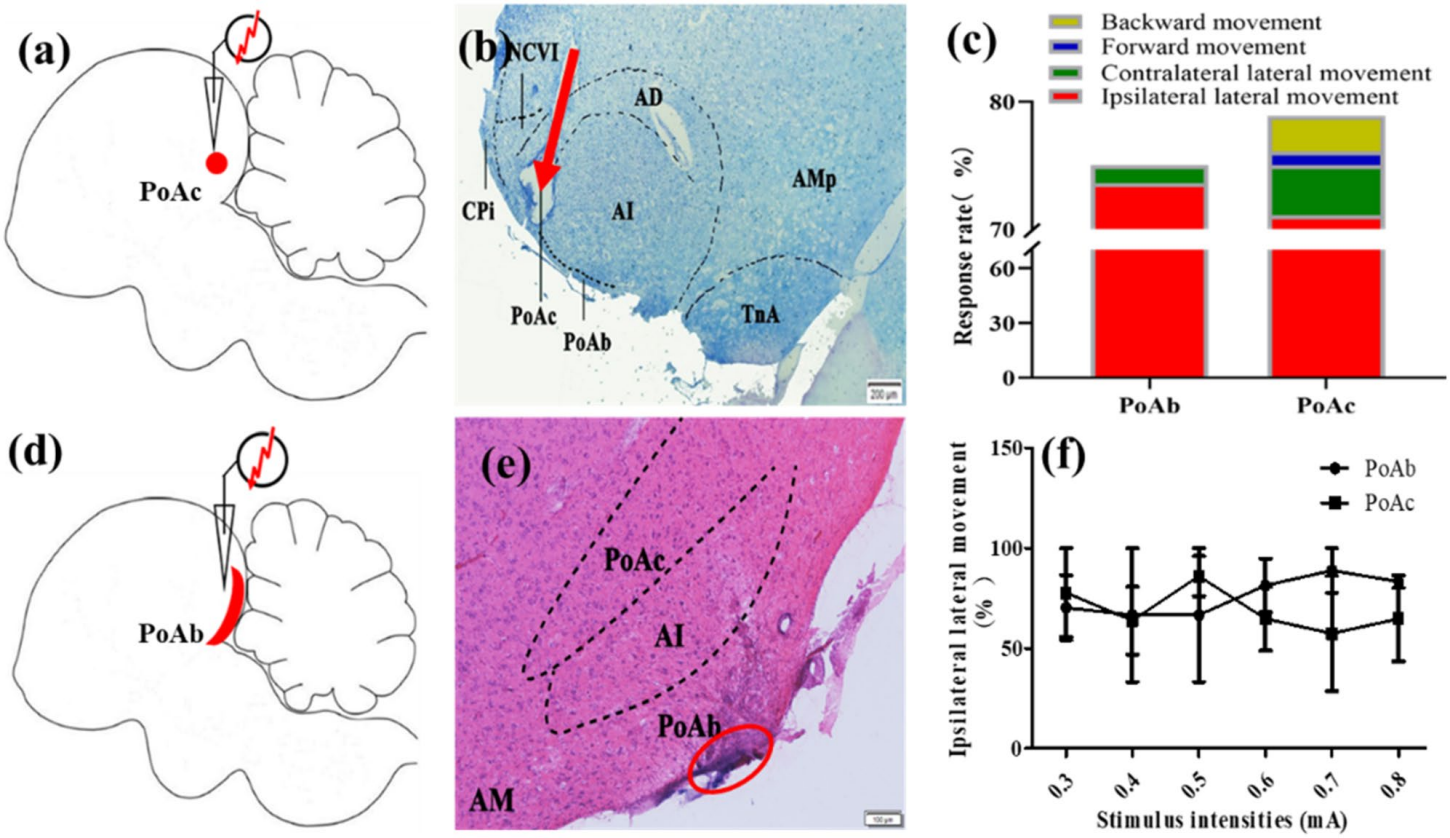
Effects of electrical stimulation on motor behaviors. Schematic sagittal view of the brain of electrical stimulation of PoAc (**a**) and PoAb (**d**). (**b**) Coronal slice of PoAc Nissl staining at A 5.0. The red arrow indicates the electrode implantation needle track. (**c**) Response rate of four different behaviors after electrical stimulation of PoAc and PoAb. (**e**) Coronal slice of PoAb hematoxylin and eosin staining at A 5.5. The red circle indicates the position of electrode tip implantation after blue dot marking. (**f**) Response rate of ipsilateral lateral movement of PoAc and PoAb at different electrical stimulation intensities. Scale bars = 200 µm in (**b**) and 100 µm in (**e**). *AD* arcopallium dorsale; *AI* arcopallium intermedium; *AM* arcopallium mediale; *AMm* arcopallium mediale, pars magnocellularis; *AMp* arcopallium mediale, pars parvocellularis; *CPi* cortex piriformis; *NCV* nidopallium caudoventrale; *PoAb* basal division of posterior pallial amygdala; *PoAc* compact division of posterior pallial amygdala; *TnA* nucleus taeniae amygdalae.

Before the electrical stimulus, the pigeons stays in place, generally accompanied by turning, or raising the head or pecking feathers in the arena. A series of new different motor behaviors were induced after the electrical stimulation of PoA, among which ipsilateral lateral movement, forward movement, contralateral lateral movement, backward movement, etc*.*, occurred more frequently (Fig. 4a,d; Supplementary Video S1–S3). The different motor behaviors elicited in the arena were defined using a previous study^12^ with slight modifications (see Table 2 for details). Figure 4c showed that PoAb mainly mediated ipsilateral lateral movement (75.00%) and seldomly mediated contralateral lateral movement (1.85%). PoAc was more complex, the behavioral response of it was as follows: ipsilateral lateral movement (78.89%), contralateral lateral movement (3.89%), forward movement (1.11%), and backward movement (2.78%). The result indicated that PoA mainly mediated the turning movement behavior in pigeons. There was no significant difference in the ipsilateral lateral movement response rate between PoAc and PoAb (Fig. 4f). The locations of the stimuli in PoAc and PoAb are presented in Fig. 4b and Fig. 4e, respectively.

**Table 2:** Definitions of some motor behaviors in the arena.

Motor behaviors	Definition of the motor behaviors
Ipsilateral lateral movement	Alternate stepping of both feet towards right side of the body
Forward movement	Alternate stepping of both feet and moving forward
Contralateral lateral movement	Alternate stepping of both feet towards left side of the body
Backward movement	Alternate stepping of both feet and moving backward

The number of steps generated by different behaviors is generally 2–3.

### The effects of different drugs on forward behavior

#### Personalized screening

**Figure 5:**
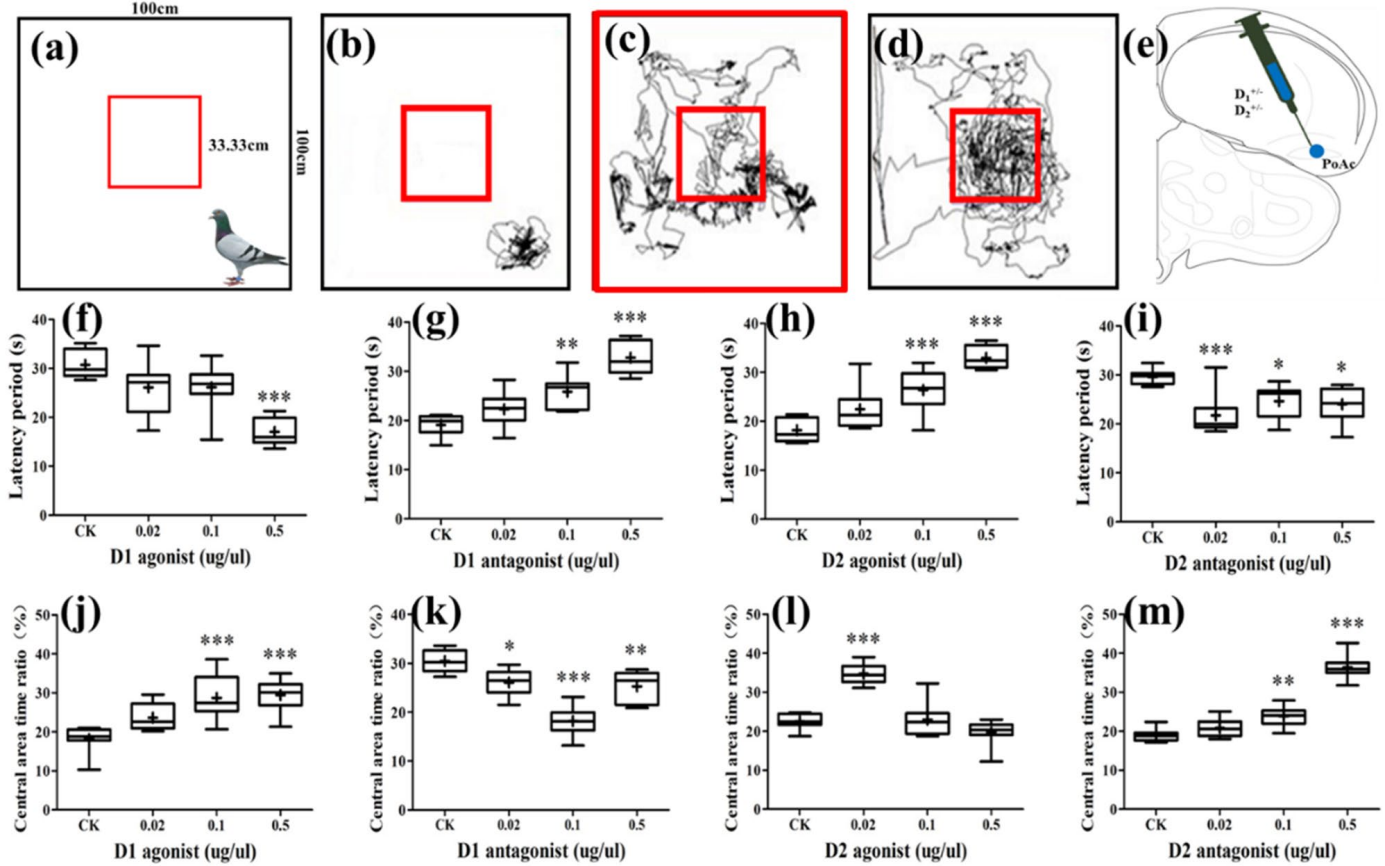
Effects of different drugs on forward behavior. (**a**) Schematic of personalized screening (*N* = *25*). (**b**–**d**) Locomotion trajectory of different pigeons (**b**: *N* = *8,* **c**: *N* = *8,* **d**: *N* = *9*). (**e**) Schematic coronal view of in vivo injections of different drugs into PoAc at A 5.0. (**f**–**i**) Effects of D1 and D2 receptor agonists and antagonists on the latency period of pigeons for entering the central area (*N* = *8*). (**j**–**m**) Effects of D1 and D2 receptor agonists and antagonists on the residence time of pigeons in the central area (*N* = *8*). Data of all groups are presented as mean ± SE. *PoAc* compact division of posterior pallial amygdala; *SE* standard error of mean.

According to the SMART output data, the total scores of 25 pigeons were obtained. From Fig. 5, we can see that eight pigeons did not enter the central area (the total score < 0.9, among which the total score was 0 was the most) (Fig. 5b), and nine pigeons were very active in the central area (the total score was > 1.50, Fig. 5d). Combining the locomotion trajectories of the pigeons in the experimental box captured by SMART, we finally selected those individuals with the total scores between 0.90 and 1.50 (Supplementary Fig. S1-S4). Consequently, eight pigeons were used for the drug injection experiment (Fig. 5c,e).

#### The effects of D1 and D2 receptor agonists and antagonists on the latency of entering central area in pigeons

The effects of D1 and D2 receptor agonists and antagonists on the latency period of pigeons entering the central area were different (Fig. 5). D1 agonist decreased the latency period of pigeons for entering the central area (F_3, 28_ = 14.78, *P* < 0.001; Fig. 5f). When the concentration of D1 agonist was 0.5 μg/μL, the range of the latency period of pigeons for entering the central area decreased by 44.61% (*P* < 0.001), but there was no significant difference in the other groups. In contrast, D1 antagonist increased the latency period of pigeons for entering the central area (F_3, 28_ = 27.58, *P* < 0.001; Fig. 5g). When the concentration of D1 antagonist was 0.1 μg/μL and 0.5 μg/μL, the latency period increased significantly, and their ranges increased by 35.46% (*P* < 0.01) and 71.61% (*P* < 0.001), respectively. For 0.02 μg/μL concentration of D1 antagonist, the latency period did not increase significantly. D2 agonist also increased the latency period of pigeons for entering the central area (F_3, 28_ = 25.63, *P* < 0.001; Fig. 5h). The ranges of the latency period increased by 45.24% (*P* < 0.001) and 81.40% (*P* < 0.001) when the concentrations of D2 agonist were 0.1 μg/μL and 0.5 μg/μL, respectively. However, 0.02 μg/μL D2 agonist exhibited no significant influence. D2 antagonist decreased the latency period of pigeons for entering the central area (F_3, 28_ = 7.74, *P* < 0.001; Fig. 5i). The latency period of pigeons for entering the central area was decreased by 26.46% (*P* < 0.001) when the concentration of D2 antagonist was 0.02 μg/μL; but when the concentrations of D2 antagonist were 0.1 μg/μL and 0.5 μg/μL, the latency period was decreased by 16.69% (*P* < 0.05) and 18.89% (*P* < 0.05), respectively. Overall, D1 agonist and D2 antagonist can reduce the latency period of pigeons for entering the central area. In contrast, D1 antagonists and D2 agonists could increase the latency period of pigeons for entering the central area.

#### The effects of D1 and D2 receptor agonists and antagonists on the residence time of pigeon in the central area

D1 and D2 receptor agonists and antagonists have different effects on the residence time of pigeons in the central area (Fig. 5). D1 agonist increased the residence time of pigeons in the central area (F_3, 28_ = 11.59, *P* < 0.001; Fig. 5j). When the concentration of D1 agonist was 0.1 μg/μL and 0.5 μg/μL, the residence time of pigeons in the central area increased by 58.12% (*P* < 0.001) and 62.03% (*P* < 0.001), respectively, but when the concentration of D1 agonist was 0.02 μg/μL, no significant difference was noted in the residence time. D1 antagonist decreased the residence time of pigeons in the central area (F_3, 28_ = 26.19, *P* < 0.001; Fig. 5k). The residence time of pigeons in the central area decreased by 40.21% (*P* < 0.001) when the concentration of D1 antagonist was 0.1 μg/μL. D2 agonist had a significant impact on the residence time of pigeons in the central area (F_3, 28_ = 34.64, *P* < 0.001; Fig. 5l). When the concentration of D2 agonist was 0.02 μg/μL, the residence time in the central area increased by 53.72% (*P* < 0.001); whereas it decreased by 12.53% when the concentration of D2 agonist was 0.5 μg/μL, and no significant difference was noted. D2 antagonist increased the residence time of pigeons in the central area (F_3, 28_ = 81.74, *P* < 0.001; Fig. 5m). The residence time of pigeons in the central area increased by 25.92% (*P* < 0.01) and 92.19% (*P* < 0.001) when the concentration of D2 antagonist was 0.1 μg/μL and 0.5 μg/μL, respectively; however, 0.02 μg/μL D2 antagonist did not result in significant changes in the residence time. In summary, with increase in drug concentration, D1 agonist and D2 antagonist significantly increased the residence time of pigeons in the central area. In contrast, D1 antagonist decreased the residence time of pigeons in the central area. Further, D2 agonist increased the residence time of pigeons in the central area at a low concentration but decreased the residence time at a high concentration.

## Discussion

The present study revealed the fiber connections of PoAb, which innervate most of the telencephalon, especially the pallium. Concerning descending pathways, PoAb projects primarily to the midbrain. The labeling areas are consistent with those reported by previous researchers in forebrain^6,8,9,13–18^. Compared with PoAb, PoAc has few connections with the pallium. As for the downstream pathways, PoAc connects primarily with the diencephalon and projects less on the lateral hypothalamus. The connections between PoAb and pallium were confirmed in pigeons, which are in accordance with the findings in chickens or mallards, although of the projections between PoA and the ventral basal ganglia or medial striatum have not been proven in chickens or mallards^19,20^. While previous studies of tracing studies about the tectal layers and the associated isthmic complex were not described ascending projections to the pallial. But the present study revealed that PoAb connects reciprocally with SGC, SGF, and SAC and tectal layers. The connectivity of PoAb was a mixture of different subjects, which may be caused by partial leakage of CTB or BDA in some individuals. Arcopallium belongs to the premotor brain area, in which AM, AD and AI can also stably mediated the generation of motor behavior in pigeons after electrical stimulation (unpublished results). Therefore, there is a close connection between Arcopallium and many of the midbrain motor brain area, although the fiber connection of Arcopallium has not yet been reported. The presence of fiber connections with part of the midbrain area may be due to the leakage of part of the tracer into arcopallium. Moreover, this may be due to different color rendering methods causing certain differences in results, also it may be caused by some false positive results. Thus we did not draw the controversial brain areas in the summary diagram. As the second major vertebrates, birds have no corpus callosum (CC). The connection of PoAb with many areas in the brain of pigeons may suggest that it plays an essential role in interhemispheric exchange.

The PoA, TnA, area subpallialis amygdalae (SpA), bed nucleus of the stria terminalis, and pars lateralis (BSTL) were considered to form the avian amygdala^7^. The mammalian amygdala initiates predation behavior and affects the expression of appetite behavior^21,22^. Till date, limited studies have examined the role of the amygdala in the expression of motor behaviors in birds, and existing studies have shown that the amygdala mediate distinct aspects of appetitive extinction learning^23^, and TnA could induce right and left body turns in pigeon^24^. The role of PoA in the regulation of motor behavior is not clear. Therefore, it is useful to study the function of PoA. Before the electrical stimulus, the pigeons stays in place in the arena, but they would respond immediately after being electrically stimulated. The results indicated that PoAb can mainly mediate ipsilateral lateral movement, whereas PoAc is different. The differences in PoAb and PoAc with regard to connectivity result in differences in their mediation of motor behaviors. PoAb has closer connections with pallium than does PoAc. As for the descending pathways, PoAb connects with many locomotor brain regions in the midbrain, whereas PoAc has little connections with locomotor brain regions in the midbrain, most of which are one-way projections. PoAc has significantly more connections with other brain regions within the amygdala of birds than does PoAb^8^. Perhaps the close connections within the amygdala lead to the complexity of PoAc behavior. Moreover, the molecular mechanisms of PoAb and PoAc on the mediation of motor behavior in pigeons remain to be studied.

Each pigeon’s total score exhibited whether the activity time and activity degree of the pigeon in the central area were random or not. The trajectories of the 24 pigeons were divided into three parts as shown in the Fig. 5b–d, respectively. The individuals in Fig. 5b represent extremely inactive pigeons, which were cautious subjects. The individuals in Fig. 5d represent the hyperactive subjects, which were reckless pigeons. Neither types of pigeons met the requirements of our subsequent experiments. In order to clearly show the difference of different results in subsequent drug injection experiments, we selected the neutral individuals in Fig. 5c. To eliminate the effects of stress or fear, the pigeons were allowed 5 min to acclimate to the new environment, and the next 10 min were the formal screening. Similar to mammal, birds generally tend to move in the edge area in an unfamiliar environment, and rarely enter the central area. Only after being familiar with the surrounding environment, they would random movements appear in the central and edge areas. This adaptation was formed in the long-term evolution of animals.

The effects of different drugs on forward behavior revealed that D1 agonist and D2 antagonist could promote forward behavior, whereas D1 antagonist and D2 agonist have inhibitory effects. Our results corroborated those of previous studies in Wistar rats, hens, and Japanese quail^25–28^. However, compared with reptiles, the effects of D1 and D2 receptor agonists and antagonists were partly in accordance with those on pigeons^29,30^; this could be attributed to the difference in species. As far as the fiber connection of PoAb was concerned, we thought it may play an important role in motor behavior, but the electrical stimulation results show that PoAb brain region regulation behavior was relatively simple while PoAc was relatively complex. Since experiments in this part mainly analyze the changes of pigeons in behavior before and after injection of different drugs, the forward behavior was directly related to the pigeon movement. Besides, tyrosine hydroxylase as a marker of dopaminergic fibers in the avian telencephalon^31^, PoAc was characterized by high tyrosine hydroxylase (TH) immunoreactivity, whereas PoAb has negligible numbers of TH positive cells^7,9^. Thus, we chose the dominant site of forward behavior in PoAc for drug injection. Existing studies have shown that dopamine plays an important role in regulating fear and anxiety^32–34^, PoA was an important brain area for emotion regulation^8^, and micro-injection of drugs can lead to behavioral changes due to emotional changes in animals. By comparing the behavioral differences between the experimental group and the control group, the role of PoAc in the regulation of forward behavior is studied. The results of this part may indicated that PoAc affects the behavior of pigeons by regulating their emotions.

The stainless steel stimulation electrode was used in this study and only the tip discharge, while histological verification shows that the electrode tips were located in the target brain area. All electrical stimulation parameters and experimental procedures were in line with the requirements of medical ethics. Later long-term observations demonstrated that the pigeons implanted with electrodes lived normally like their peers and still maintained normal reproduction ability, and their motor ability and lifespan were not affected. Studies on the treatment of epilepsy and Parkinson disease through deep brain stimulation were also common in human brain diseases^35–37^.

Overall, PoAb connects with many more areas in the cerebrum than PoAc, may suggest that the role of PoAb was exchanged between the right and left hemispheres. The function of PoAb and PoAc is to mediate the turning behavior, and both of them can be used as important brain areas to regulate turning behavior. The effects of different drugs on forward behavior may indicated that PoAc affects motor behaviors by regulating emotion. Our study provides a theoretical basis for controlling motor behavior in birds and enriches the theoretical system of emotion-related brain regions and behaviors in birds.

## Methods

### Animals

Overall, 65 adult pigeons (body mass, 350–450 g) were used in this study. All animals (unknown sexes) were acclimatized to a wire cage (95 × 80 × 65 cm^3^) under a normal day/night light cycle with food and water ad libitum. The feedstuff was composed of wheat, corn, sorghum, and soybean at the ratio of 1:1:1:1, and the cages were cleaned twice a week. All experimental procedures and animal housing and manipulations were approved by and carried out under the Life Science Ethical Review Committee of Zhengzhou University (Henan, China). All efforts were made to minimize the suffering of the animals throughout the experimental procedures.

#### Declaration

The study was carried out in compliance with the ARRIVE guidelines, and all methods were carried out in accordance with relevant guidelines and regulations.

### Tract-tracing of PoAb pathway

#### Surgical procedure

The pigeons were anesthetized by injecting them with 3% Pelltobarbitalum Natricum (0.12 ml/100 g body mass) intraperitoneally. After the pain reflex disappeared, the pigeons’ heads were placed in a stereotaxic holder customized for the pigeons. The skin above the surgical field was removed carefully, part of the skull was removed using a dental drill, the dura mater and arachnoid mater were moved, and the brain tissue was exposed. Subsequently, the injection cannula (O.D.0.41 × I.D.0.25; R.W.D. Life Science, Shenzhen, China) was implanted. When the PoAb was reached, the gaps between the cannula and the cranium were sealed with EC glue and fixed with dental cement after the glue solidified. Enrofloxacin solution (5%) was used in the surgical area to help recovery. The surgery area was cleaned regularly. The pigeons were fed separately during 5–7 days of recovery, after which they were used in the following experiments.

#### In vivo* injections*

After recovery, each pigeon received unilateral injections of one tracer, BDA (10,000 molecular weight; Thermo Fisher, Eugene, OR, USA) or CTB (low salt; List Biological Laboratories, Campbell, CA, USA) on the right hemisphere (Fig. 1a,i). BDA (10% in phosphate-buffered saline [PBS], pH 7.4) was used for anterograde labeling, whereas CTB (1% in PBS) was used for retrograde labeling. At each injection site, 150 nl of the tracer was applied at each depth using a mechanical pressure device (R.W.D. Life Science, Shenzhen, China). After 2 days (for CTB injections) or 7 days (for BDA injections) of survival, the pigeons were deeply anesthetized using Pelltobarbitalum Natricum (100 mg·kg^−1^) and perfused with physiological salt solution, followed by cardiac perfusion with 4% formaldehyde. Their brains were extracted and postfixed overnight in 4% paraformaldehyde at 4 °C and subsequently transferred to 30% sucrose in PBS at 4 °C for 3 days. Brains were cut at the coronal plane in 35-µm thick slices using a freezing microtome (Leica CM1950, Germany).

#### Immunohistochemistry

BDA and CTB fluorescence staining was used to demonstrate anterogradely labeled fibers and retrogradely labeled neurons.

For CTB fluorescence staining, slices were washed with PBS before (3 × 10 min) and after (1 × 5 min) incubation in 10% normal goat serum (30 min; 1:10 in PBS). Subsequently, they were incubated with polyclonal rabbit anti-CTB antibody (Abcam, RRID: AB_34992; 1:1,000 in PBS) for 12–14 h at 4℃. Then, the slices were washed in PBS (3 × 10 min) and incubated with the secondary antibody Alexa Fluor 488-conjugated AffiniPure Goat Anti-Rabbit IgG (Wuhan service biotechnology; 1:200 in PBS) for 1 h at 37℃. Finally, the slices were washed in PBS (3 × 10 min), mounted on glass slides, and covered using a coverslip with fluoromount (Wuhan service biotechnology, Wuhan, China).

For BDA fluorescence staining, after the slices were incubated for 30 min in 10% normal goat serum, they were incubated with Alexa Fluor 594 streptavidin (BioLegend Way San Diego, CA; 1:200 in PBS) for 50 min at 37℃. Finally, the slices were washed in PBS (3 × 10 min), mounted on glass slides, and covered using a coverslip with fluoromount.

### The effects of PoA electrical stimulation on motor behaviors

#### Microelectrode implants

The electrode (diameter = 120 μm; stainless steel wire, Teflon insulation) was implanted into PoAb and PoAc, respectively (*N* = 3). All processes were performed as described above.

#### Electrical stimulation protocols

After recovery, the behavioral responses of the pigeons to electrical stimulation were observed. The subjects were given 5 min for adaptation, and then electrical stimulation (stimulation intensity = 0.3–0.8 mA; the interval between different stimulation intensities was 3–5 min; frequency = 20 Hz) generated by an YC—2—S bipolar programmed stimulator (Chengdu Instrument Factory, China) was applied via stainless steel electrodes (diameter = 120 μm; impedance range < 0.5 MΩ). The test was done in an arena made of polymathic methacrylate (diameter × height, 80 × 80 cm^2^). The responsive behaviors evoked by different stimulation intensities were recorded using a SONY HDR-CX220 camcorder (Sony Corporation, Tokyo, Japan). By comparing the behavior changes before and after electrical stimulation, the study determined the role of PoA in the expression of motor behavior in pigeons.

#### Histology

After three test trials (inter-experiment interval was 3 days), the pigeons were deeply anesthetized and then perfused. Subsequently, their brains were removed from the skulls, postfixed, and dehydrated. Lastly, histological analyses were performed by Nissl and hematoxylin and eosin staining. Light microscopy was used to identify the stimulation sites of the sections.

### The effects of different drugs on forward behavior

#### Personalized screening

The pigeons were screened to avoid the impact of personalized differences before the experiment. According to the method used for studying jungle crow’s response to mirror-image stimulation^38^, the size of the experimental box was 100 × 100 × 40 cm^3^ (length × width × height), and the video equipment was installed 120 cm above the experimental box. The experimental box was divided into two areas: the central area (within the red box) and the edge area (outside the red box). The central area was square with an area of 33.33 × 33.33 cm^2^, accounting for 1/9 of the whole area.

The pigeons were placed in the edge area of the experimental box when the recording device was working. Each pigeon was recorded for 15 min. The first 5 min recorded the pigeons’ adaptation process to the experimental box, and the last 10 min constituted the actual personalized screening videotape. Subsequently, 75% alcohol was used to spray the whole experimental box, and the next experiment was recorded after 30 min. SMART 3.0 Panlab video analysis software (Henan Baiquan Bioscientific Co.,limited, China) was used to collect the data on movement behavior of each pigeon (*N* = 25) automatically; the data mainly included movement time, movement speed, and movement distance of the pigeon in various areas of the experimental box; the movement trajectory of the pigeon was also captured. The personality screening criteria were determined by the movement time, distance, and activity degree in each area of the experimental box. The personality score of each pigeon was calculated as follows: (movement distance in the central area × 9 / total distance) / (movement time in the central area × 9 / total time). The pigeon motion trajectory map obtained by SMART software was used on an auxiliary basis.

#### Stereotaxic implantation of the injection cannula

After personalized screening, the pigeons (*N* = 8) were implanted with an injection cannula (O.D.0.41 × I.D.0.25) at the site of PoAc (A 5.00 mm; L 7.60 mm). All processes were performed as described above.

#### Drug treatments

After cannula implantation, the recovered pigeons were administered with four different drugs (*N* = 8): SKF-38393 (D1 + : D1 agonist), SCH-23390 (D1 -: D1 antagonist), quinpirole (D2 + : D2 agonist), and raclopride (D2 -: D2 antagonist); all drugs were bought from Sigma-Aldrich (St louis, USA). According to the methods used in Japanese quail^25^ and pigeon^39^, three concentration gradients were set for each drug: 0.02 μg/μL (low), 0.1 μg/μL (medium), 0.5 μg/μL (high); 0.9% saline was used as the control. Because the four drugs can be metabolized autonomously in pigeons, two injections can be performed for each drug.

First, the pigeons in the control group were injected 1 μL of saline, and their movement behaviors were recorded in the experimental box for 30 min; then the pigeons were injected 1 μL of 0.02 μg/μL D1 agonist (low concentration), and their movement behaviors were recorded for 30 min after 15 min of drug absorption time. After 1 day, 1 μL of saline was injected as control, following which 1 μL of 0.1 μg/μL D1 agonist (medium concentration) was injected; after 15 min, their movement behaviors were recorded for 30 min. Another day later, 1 μL of saline was injected as control, and then 1 μL of 0.5 μg/μL D1 agonist (high concentration) was injected; after 15 min, their movement behaviors were recorded for 30 min. Different concentrations of D1 antagonist, D2 agonist, and D2 antagonist were injected; the experimental procedure was the same as that for D1 agonist, and the interval between the injection of different drugs was 4 days.

### Data recording and analysis

The terminology used in the present study is based on the Avian Brain Nomenclature Forum^1,11^.

Based on the results of Nissl staining, we draw a vector coronal brain map of the whole pigeon brain (unpublished), and then overlaid the results of immunofluorescence on different vector brain maps to determine the labeled brain regions. Photomicrographs were captured using a digital camera (Olympus, Japan). The layout of micrographs, lettering, and printing, and schematic illustrations of labeled neurons and fibers were performed using Adobe Photoshop 7.0 J (Tokyo, Japan) and Adobe Illustrator 10.0J (Tokyo, Japan).

All data were analyzed using IBM SPSS Statistics 24 (Chicago, USA). Data were recorded using SMART 3.0 software, which included all relevant data and animal behavior trajectory data. The data standardization method was as follows: for each animal, (data of each treatment/sum of four treatments) × 100%. One-way ANOVA was used to analyze the drug administration data. Data of all groups are represented as mean ± standard error of the mean.

## Supplementary Information

Supplementary Legends.

Supplementary Video 1.

Supplementary Video 2.

Supplementary Video 3.

Supplementary Figure S1.

Supplementary Figure S2.

Supplementary Figure S3.

Supplementary Figure S4.
