# Supplementary material for: The role of posterior pallial amygdala in mediating motor behaviors in pigeons
Source: Sci Rep. 2022 Jan 10;12:367. doi: 10.1038/s41598-021-03876-7 (PMC8748633; doi:10.1038/s41598-021-03876-7)
Supplement: Supplementary file 1 — Supplementary Legends. [file 41598_2021_3876_MOESM1_ESM.docx]

Supplementary Data:

**Supplementary** **Figures**

**Supplementary Figure S1 –** The trajectory of the pigeon No. 06 after being injected with normal saline.

**Supplementary Figure S****2 –** The trajectory of the pigeon No. 06 after being injected with 0.02μl D1+.

**Supplementary Figure S3 –** The trajectory of the pigeon No. 06 after being injected with 0.1μl D1+.

**Supplementary Figure S4 –** The trajectory of the pigeon No. 06 after being injected with 0.5μl D1+.

**Supplementary** **Videos**

**Supplementary Video 1** –Behavioral changes of the pigeon No. 080 before and after first electrical stimulation at 0.7mA.

**Supplementary Video 2** –Behavioral changes of the pigeon No. 080 before and after second electrical stimulation at 0.7mA.

**Supplementary Video 3** – Behavioral changes of the pigeon No. 080 before and after third electrical stimulation at 0.7mA.
