# Supplementary figures and images for: The role of posterior pallial amygdala in mediating motor behaviors in pigeons
Source: Sci Rep. 2022 Jan 10;12:367. doi: 10.1038/s41598-021-03876-7 (PMC8748633; doi:10.1038/s41598-021-03876-7)

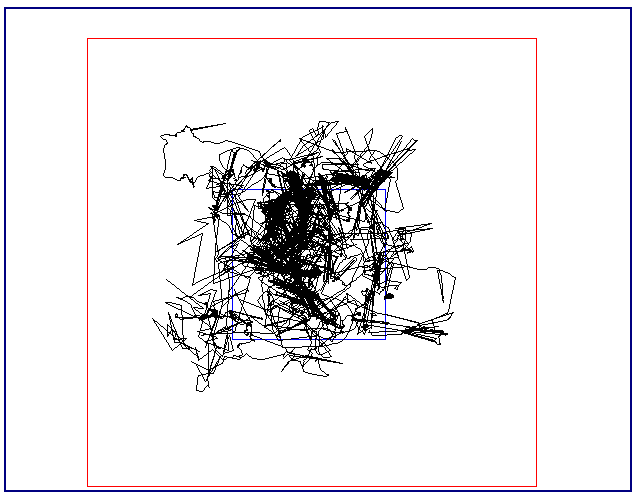

Supplement: Supplementary file 5 — Supplementary Figure S1. [file 41598_2021_3876_MOESM5_ESM.bmp]

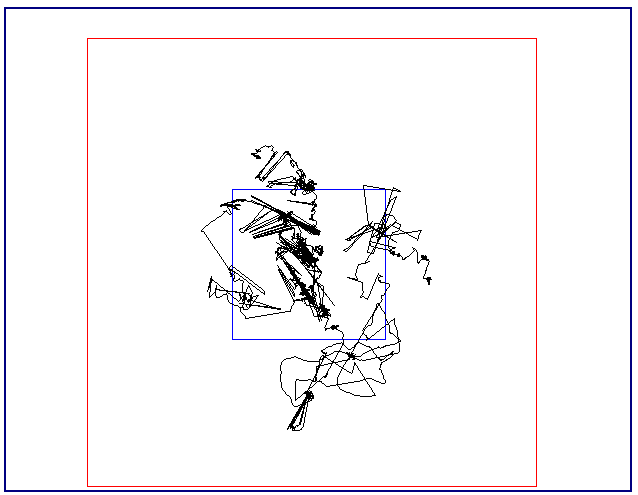

Supplement: Supplementary file 6 — Supplementary Figure S2. [file 41598_2021_3876_MOESM6_ESM.bmp]

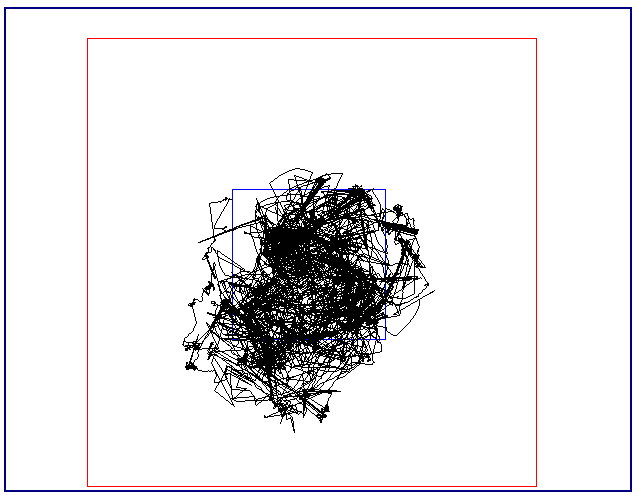

Supplement: Supplementary file 7 — Supplementary Figure S3. [file 41598_2021_3876_MOESM7_ESM.bmp]

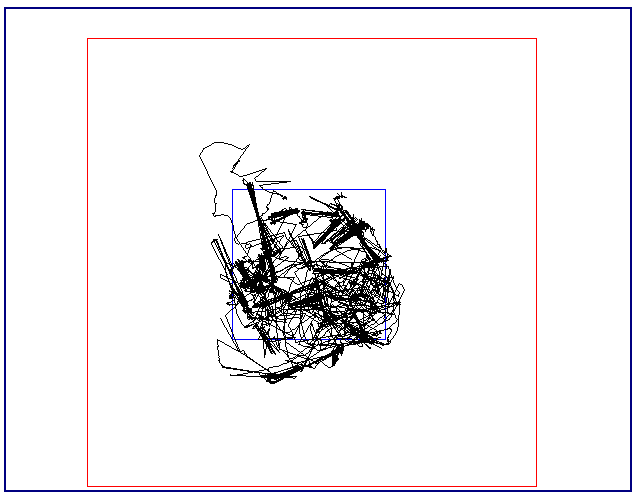

Supplement: Supplementary file 8 — Supplementary Figure S4. [file 41598_2021_3876_MOESM8_ESM.bmp]
